# C-reactive protein predicts complications in community-associated *S. aureus* bacteraemia: a cohort study

**DOI:** 10.1186/s12879-021-05962-7

**Published:** 2021-04-01

**Authors:** Carly L. Botheras, Steven J. Bowe, Raquel Cowan, Eugene Athan

**Affiliations:** 1 School of Medicine, IMPACT, the Institute for Mental and Physical Health and Clinical Translation, Deakin University, Geelong, Australia; 2 School of Medicine, Faculty of Health, Deakin University, Geelong, Australia; 3 Deakin Biostatistics Unit Faculty of Health, Deakin University, Geelong, Australia; 4 Department of Infectious Diseases, Barwon Health, Geelong, Australia

**Keywords:** *S. aureus* bacteraemia, Complications, Epidemiology, C-reactive protein

## Abstract

**Background:**

*Staphylococcus aureus (S. aureus)* bacteraemia is increasingly acquired from community settings and is associated with a mortality rate of up to 40% following complications. Identifying risk factors for complicated ***S. aureus*** bacteraemia would aid clinicians in targeting patients that benefit from expedited investigations and escalated care.

**Methods:**

In this prospective observational cohort study, we aimed to identify risk factors associated with a complicated infection in community-onset *S. aureus* bacteraemia. Potential risk factors were collected from electronic medical records and included: - patient demographics, symptomology, portal of entry, and laboratory results.

**Results:**

We identified several potential risk factors using univariate analysis. In a multiple logistic regression model, age, haemodialysis, and entry point from a diabetic foot ulcer were all significantly protective against complications. Conversely, an unknown entry point of infection, an entry point from an indwelling medical device, and a C-reactive protein concentration of over 161 mg/L on the day of admission were all significantly associated with complications.

**Conclusions:**

We conclude that several factors are associated with complications including already conducted laboratory investigations and portal of entry of infection. These factors could aid the triage of at-risk patients for complications of *S. aureus* bacteraemia.

## Background

*S. aureus* is a Gram-positive bacterium that can cause many diseases including bacteraemia. Cases of *S. aureus* bacteraemia [SAB] are common and incidence rates remain stable [1, 2]. In Australia, SAB occurs at an annual rate of 10 per 100,000 people [3] and is mostly acquired from the community [4]. Risk of mortality remains high with international data suggesting rates of up to 40% [5] with Australian mortality currently reported at 18.3% [3]. Complications of SAB are common and include infective endocarditis, osteomyelitis, and severe sepsis. These complications are responsible for the need of greater interventions, longer admissions to hospital and increased risk of mortality.

Several studies have aimed to identify specific factors that may aid clinicians in identifying complications in SAB [6–8]. There is debate on gender, age and co-morbidity burden, with increased incidence of infection noted, but polarising results on outcome [9–11]. The antibiotic resistance profile of *S. aureus* [12], whether it persists with treatment [13], and where it is acquired are also possible risk factors for complications [11].

Predictive biomarkers have also been studied. C-reactive protein [CRP] is an acute phase reactant released from the liver and is raised in response to tissue injury and metabolic stressors. Several conditions that increase CRP have been observed including inflammatory processes, cancer, pregnancy and infection [14–16].

Although CRP has been rejected as a diagnostic marker for SAB due to the lack of specificity [17], it has also been investigated as a predictive factor of SAB complications. There are limitations to extrapolating these results to a clinical setting. Firstly, international studies on SAB often include healthcare-acquired infections and do not accurately reflect the increasingly common community-associated setting [18]. Other studies do not focus solely upon *S. aureus*, limiting the translation of the findings [19]. It is important to identify factors, including CRP, that are associated with an increased risk of complicated SAB acquired from the community. This is to better inform management guidelines and impact on mortality rates.

In this study, we aimed to identify potential predicting factors for complication of community-associated SAB infections from all adult cases of SAB that presented to a single institution in Australia. We ascertained several factors that could predict complications and suggest that CRP may be a very useful and readily available biomarker.

## Methods

### Sample selection

**Fig. 1:**
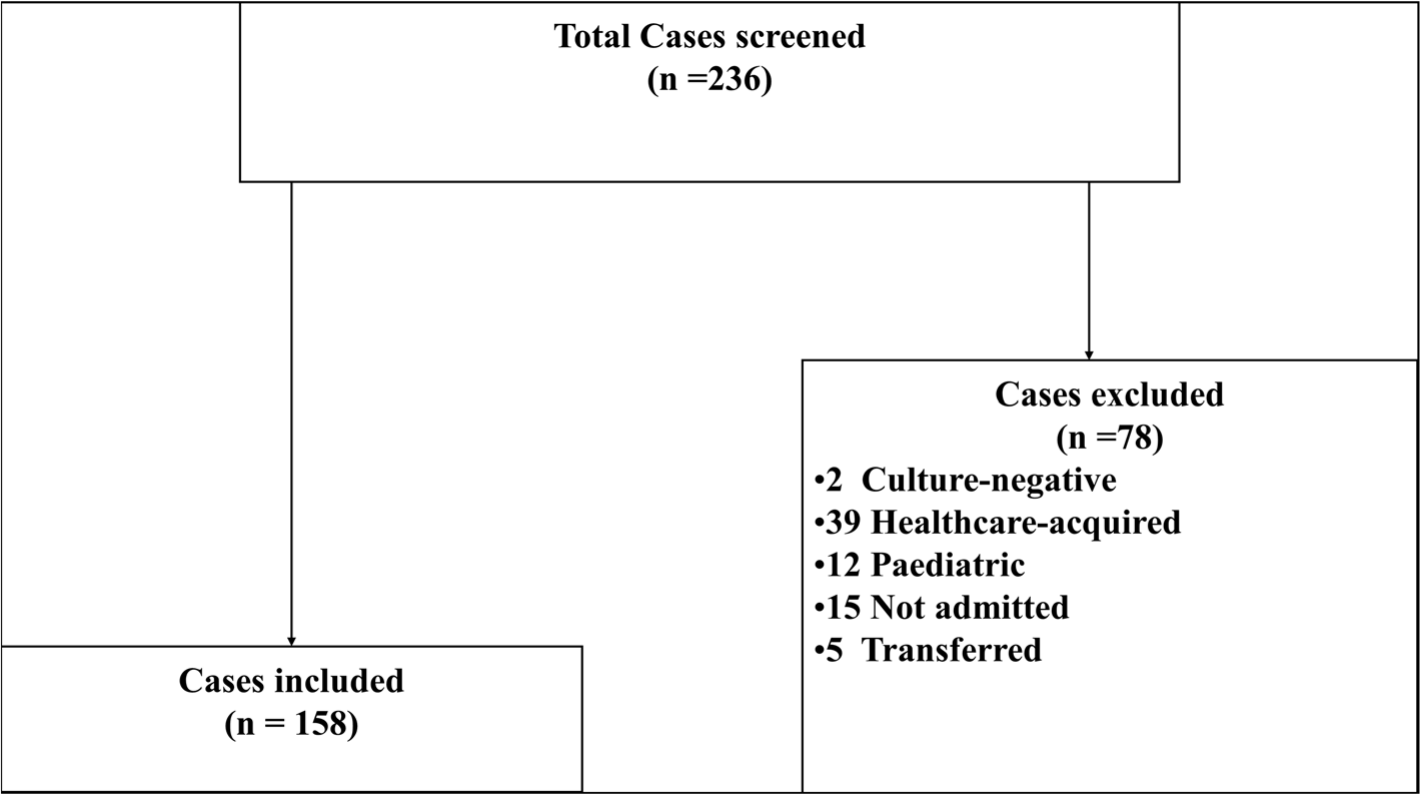
.

University Hospital Geelong [UHG] is a 500-bed tertiary referral hospital in the south-west of Victoria, Australia. It has a catchment of a population of 600,000 and has a specialised Infectious Diseases service. From June 2015 to September 2018, 236 adult cases of SAB were prospectively identified at UHG. All adult cases admitted to the UHG with an initial positive blood culture of *S. aureus* flagged within 48 h of admission to hospital were included into the study (Fig. 1). Paediatric cases, culture negative cases, or a blood culture positive after 48 h of admission to hospital [healthcare-acquired] were excluded. Cases not admitted to UHG or transferred to another hospital before a complication was diagnosed were also excluded.

### Categorisation

Cases included into the study were categorised on whether they were an uncomplicated or complicated. Categorisation were based upon definitions from the Infectious Disease Society of America Definition [20]. This defines uncomplicated SAB as a SAB which has no infective endocarditis or metastatic infection, no presence of an indwelling medical device within the patient, defervescence within 72 h of antibiotic commencement and no positive blood cultures after 72 h following commencement of antibiotics. Complicated SAB was classified as cases of SAB which did not match the uncomplicated definition.

### Data collection

Cases were prospectively identified through the microbiology service (Australian Clinical Laboratories) and clinical data was collected from electronic medical records [EMR]. Data collected included patient demographics, symptomology and portal of entry, microbiological details, and biomarker and blood cell counts from the day of admission. These variables were selected as routinely collected data and their potential as predictive variables for SAB complications. These factors are further detailed below.

### Demographics

Demographics included age, gender and co-morbidities. Co-morbidity burden was determined through the Charlson age co-morbidity index [CACI] [21]. The CACI considers age, HIV status, renal and liver function, diabetic status, and peripheral vascular conditions as well as other conditions [21]. Other co-morbidities not used in the CACI, such as injecting drug use, immunosuppressive status, skin conditions and haemodialysis were also collected, as these co-morbidities are frequently associated with *S. aureus* infections [22–26].

### Symptomology and portal of entry

Symptoms were described on admission and recorded in the EMR by the admitting medical officer. These included fever, rigors, nausea, lethargy, and pain. Pain included both local and generalised pain.

Portal of entry was defined as previously described from Smit et al. [27] with a few modifications. Briefly Smit et al. lists skin and soft tissue infection [SSTI], indwelling medical device associated [IMD], injecting drug use inoculation [IDU], other injections, other entry points, or unknown [27]. We did not include other injections and other entry points as we only had two other entry points observed and no other injections were observed. We instead included community-acquired pneumonia and separated diabetic foot ulcer from SSTI due to the potential increases risk of poor outcomes from these entry points [5, 28, 29]. SSTIs included pressure ulcers, non-diabetic foot ulcers, cellulitis, and other infections of the skin and soft tissue such as abscesses and boils. Pneumonia was defined as community-acquired pneumonia where there were no other entrance points of infection. IMD included catheters and recent implanted devices. IDU was defined as a SAB infection directly attributable to injecting drug use. An unknown entry point was defined as a case that didn’t have any defined entry point.

### Laboratory test factors

Microbiological data included whether the bacteraemia was persistent, as defined as having three or more days of consecutive positive blood cultures. The antibiotic sensitivity of the organism was determined through the VITEK 2 system [Biomerieux] and cefoxitin discs and were classified via definitions used by the pathology services. Those pan-sensitive were designated as penicillin-sensitive [PSSA], isolates resistant to penicillin were classified as methicillin-sensitive [MSSA], those resistant toward cefoxitin but no other classes were classified as non-multi resistant [nm-MRSA] and those resistant to cefoxitin and other antibiotic classes were classified as methicillin-resistant [MRSA]. It was also noted if the SAB was part of a poly-microbial infection.

Biomarkers and blood tests were ordered at the discretion of the clinical team in charge of the patient on the day of admission. Results of available biomarkers and bloods were collected and included CRP concentration, platelet, leukocyte, neutrophil and lymphocyte cell counts. If a biomarker was collected more than once, the most divergent from the healthy range was chosen.

### Statistical analysis

#### Data presentation

The outcome for the study was considered as a binary variable: Complicated infections (Yes/No).The explanatory/predictor variables of interest are both continuous and categorical variables. These include demographics, self-reported symptomology, portal of entry and microbiological and laboratory assessment variables. All categorical data are presented as % [n], and non-normal continuous data are presented as medians and interquartile ranges [IQR].

#### Univariate analysis- stage 1

Pearson’s Chi squared and Fisher’s Exact tests and univariable logistic regression were used to determine whether there were associations between the outcome: complicated infections (Yes/No) and potential categorical predictor/exploratory variables. Whereas univariable logistic regression was used to explore potential associations between complicated infections (Yes/No) and continuous exploratory variables. Only results from the univariable logistic regressions are presented.

#### Receiver operating characteristic (ROC) curve analysis- stage 2

Significant blood markers determined from univariate analysis were also assessed for a potential cut-off value. The main blood marker of interest was CRP concentration. To determine a potential optimal cut-off values, Receiver Operating Characteristic (ROC) curve analysis was used and a cut-off value was predicted using the Youden Index.

#### Multiple logistic regression- stage 3

Exploratory variables were included in the multiple logistic regression modelling, if preliminary tests had a Wald statistic *p*-value < 0.2. A stepwise forward and backward elimination approach was used to model certain potential predictors while variables deemed as potential confounders by the literature were included to adjust other associations the multiple logistic regression model. Post estimation analysis such as Hosmer-Lemeshow goodness of fit test, percentage or correctly classified and area under the curve were used to assess the appropriateness of the model. Only patients with complete data were included in the logistic regression modelling. For this exploratory analysis, a level of significance (α) of 5 and 95% confidence intervals were considered appropriate and reported. Although in the discussion of the final adjusted logistic regression model, a more conservative alpha level of 0.01 has been considered. All data were analysed using both Stata SE version 15.1 [30] and SPSS version 24.0 [31].

## Results

### Description of the cohort

From 2015 until 2018, 236 cases of SAB presented to UHG. After applying exclusion criteria, 158 cases of SAB were included into the study (Fig. 1). Complicated SAB represented 67.6% (*n* = 107) cases of the study and 32.4% (*n* = 51) were categorised as uncomplicated.

### Demographics

The median age was 68.5 years old (IQR: 29), CACI scores were a median of 5 (IQR: 6) and females represented 39.2% of the cohort. There were no significant associations of CACI score or gender with presentation. Intravenous drug use was low (9.5%, *n* = 15) in the cohort. As summarised in Table 1, age was associated with complications (OR: 1.03, 95% CI: [1.01, 1.05], *p* = 0.003) and haemodialysis was inversely associated with complications (OR: 0.07, 95% CI: [0.01, 0.61], *p* = 0.016).

**Table 1:** Demographic comparison between uncomplicated and complicated SAB

Demographic	Total	Uncomplicated	Complicated	Comparison^a^		
	% [***n*** = 158]	% [***n*** = 51]	% [***n*** = 107]	Crude OR	95% CI	***p***-value
Age median (IQR)	68.5 (29.0)	59.0 (29.0)	72.0 (26.0)	1.03	(1.01, 1.05)	0.003
Gender						
Female	39.2 [62]	41.2 [21]	38.3 [41]	0.89	(0.45, 1.75)	0.731
Male	60.8 [96]	58.8 [30]	61.7 [66]			
CACI median (IQR)	5.0 (6.0)	5.0 (6.0)	5.0 (6.0)	1.03	(0.93, 1.13)	0.597
Immunosuppression	13.2 [21]	17.6 [9]	11.2 [12]	0.59	(0.23, 1.51)	0.269
Diabetes	32.3 [51]	37.3 [19]	29.9 [32]	0.72	(0.36, 1.45)	0.357
Injecting Drug User	9.5 [15]	13.7 [7]	7.5 [8]	0.51	(0.17, 1.49)	0.217
Skin conditions^b^	3.8 [6]	7.8 [4]	1.9 [2]	0.22	(0.04, 1.27)	0.090
Haemodialysis	4.4 [7]	11.8 [6]	0.9 [1]	0.07	(0.01, 0.60)	0.016

*CACI* Charlson age co-morbidity index; *OR* Odds ratio; *CI* confidence interval

^a^Univariable logistic regression model

^b^Eczema and atopic dermatitis

### Self-reported symptomology

Self-reported symptomology on admission is summarised in Table 2. Pain was the most common reported symptom (65.8%, *n* = 104), followed by fever (57.6%, *n* = 91). Lethargy was the least common reported symptom (19.6% *n* = 31). Reporting rigors was inversely associated with complications (OR: 0.49, 95% CI: [0.24, 0.99], *p* = 0.046).

**Table 2:** Symptomology comparison between uncomplicated and complicated SAB

Symptoms	Total	Uncomplicated	Complicated	Comparison^a^		
	% [***n*** = 158]	% [***n*** = 51]	% [***n*** = 107]	Crude OR	95% CI	***p***-value
Fever	57.6 [91]	58.8 [30]	57.0 [61]	0.93	(0.47, 1.83)	0.829
Rigors	32.2 [51]	43.1 [22]	27.1 [29]	0.49	(0.24, 0.99)	0.046
Nausea	38.0 [60]	47.1 [24]	33.6 [36]	0.57	(0.29, 1.13)	0.106
Lethargy	19.6 [31]	19.6 [10]	19.6 [21]	1.00	(0.43, 2.32)	0.998
Pain^b^	65.8 [104]	52.7 [32]	67.3 [72]	1.22	(0.61, 2.45)	0.574

*OR* Odds ratio; *CI* Confidence Interval

^a^Univariable logistic regression model

^b^Local and generalised pain

### Portal of entry

An unknown entry point of infection was most common, representing 36.1% (*n* = 57) of the cohort (Table 3) and was strongly associated with complications (OR: 4.54, 95% CI: [1.95, 10.57], *p* < 0.001). Conversely, entry points from SSTIs (OR: 0.35, 95% CI: [0.17, 0.71], *p* = 0.003) or DFUs (OR: 0.16, 95% CI: [0.05, 0.54], p = 0.003) were inversely associated with complications.

**Table 3:** Portal of entry compared between uncomplicated and complicated SAB

Portal of entry variables	Total	Uncomplicated	Complicated	Comparison^a^		
	% [***n*** = 158]	% [***n*** = 51]	% [***n*** = 107]	Crude OR	95% CI	***p***-value
Skin/soft tissue infections	32.9 [52]	49.0 [25]	25.2 [27]	0.35	(0.17, 0.71)	0.003
Diabetic foot ulcer	8.9 [14]	8.9 [10]	3.7 [4]	0.16	(0.05, 0.54)	0.003
Pneumonia	2.5 [4]	2.0 [1]	2.8 [3]	1.44	(0.15, 14.22)	0.754
Unknown	36.1 [57]	15.7 [8]	45.8 [49]	4.54	(1.95, 10.57)	< 0.001
Indwelling medical devices^b^	15.8 [25]	9.8 [5]	18.7 [20]	2.11	(0.75, 6.00)	0.159
Injecting drug use	3.8 [6]	3.9 [2]	3.7 [4]	0.95	(0.17, 5.37)	0.955

*OR* Odds ratio; *CI* Confidence Intervals

^a^Univariable logistic regression model

^b^Indwelling medical devices relate to any external material and include all catheter types, k-wires, prosthesis and cardiac devices

### Microbiological and laboratory assessment

As summarised in Table 4, most *S. aureus* isolates were methicillin sensitive (74.1%, *n* = 117). The prevalence of antibiotic resistant *S. aureus* was low within the study (2.5% MRSA (*n* = 4), 5.1% nm-MRSA (*n* = 8)) and was not associated with complicated SAB (*p* = 0.754 for MRSA and *p* = 0.746 for nm-MRSA). While poly-microbial *S. aureus* bacteraemia was inversely associated with complications, it was not statistically significant (OR: 0.38, 95% CI: [0.14, 1.04], *p* = 0.061).

**Table 4:** Laboratory results comparisons between uncomplicated and complicated SAB

Laboratory Result	Total	Uncomplicated	Complicated	Comparison^a^		
	% [***n*** = 158]	% [***n*** = 51]	% [***n*** = 107]	Crude OR	95% CI	***p***-value
Microbiological tests						
PSSA	18.4 [29]	17.6 [9]	18.7 [20]	1.07	(0.45, 2.56)	0.874
MSSA	74.1 [117]	74.5 [38]	73.8 [79]	0.97	(0.45, 2.07)	0.928
nm-MRSA	5.1 [8]	5.9 [3]	4.7 [5]	0.78	(0.18, 3.42)	0.746
MRSA	2.5 [4]	2.0 [1]	2.8 [3]	1.44	(0.76, 14.22)	0.754
Poly-microbial	10.8 [17]	17.6 [9]	7.5 [8]	0.38	(0.14, 1.04)	0.061
Blood tests						
C-Reactive protein concentration median (IQR)	144.0 (141.0) [139]	127.0 (129.7) [48]	160.0 (145.6) [91]	1.01	(1.00, 1.01)	0.007
Platelet cell count median (IQR)	177.5 (138.8) [150]	190.0 (126.5) [49]	175.0 (143.5) [101]	1.00	(0.99, 1.00)	0.346
White cell count median (IQR)	11.4 (7.8) [150]	10.0 (6.2) [49]	12.1 (9.5) [101]	1.07	(1.01, 1.13)	0.027
Lymphocyte cell count median (IQR)	0.8 (0.8) [150]	0.8 (0.85) [49]	0.8 (0.7) [101]	0.85	(0.54, 1.33)	0.477
Neutrophil cell count median (IQR)	9.1 (6.6) [150]	7.4 (6.2) [49]	9.8 (7.5) [101]	1.05	(0.98, 1.13)	0.146
C-reactive protein concentration over 161 mg/L	40.3 [56]	22.9 [11]	49.5 [45]	3.29	(1.50, 7.24)	0.003
White blood cell count over 14.85 × 10^9^/L	30.7 [46]	16.3 [8]	37.6 [38]	3.09	(1.31, 7.29)	0.01
Neutrophil cell count over 9.25 × 10^9^/L	48.7 [73]	36.7 [18]	54.5 [55]	2.06	(1.02, 4.15)	0.043

*PSSA* penicillin-sensitive *S. aureus*; *MSSA* methicillin-sensitive *S. aureus*; *nm-MRSA* non-multiple methicillin-resistant *S. aureus*; *MRSA* methicillin-resistant *S. aureus; IQR* interquartile range; *OR* odds ratio; *CI* Confidence interval

^a^Univariable logistic regression model

Data on blood biomarkers and from full blood examinations collected on the day of admission were analysed and compared (Table 4). Univariable logistic regressions suggested significant associations between complications and the continuous variable of CRP, white blood cell count, and neutrophil cell count. Potentially important cut-off values were determined for each of these variables using Youden’s Index and used in further analyses.

As shown in Table 4, it was observed that the presence of a CRP concentration over 161 mg/L was associated with complications (OR: 3.29, 95% CI: [1.50, 7.24], *p* = 0.003). In addition, a white cell count over 14.85 × 10^9^ cells/L (OR: 3.09, 95% CI:[1.31, 7.29], *p* = 0.01) or a neutrophil cell count over 9.25 × 10^9^ cells/L (OR: 2.06, 95% CI:[1.02, 4.15], *p* = 0.043) were also associated with complications.

### Multiple logistic regression analysis

The goodness of fit test suggested that the final multiple logistic regression model shown in Table 5 was a reasonably fitting model (*p* = 0.744). When all other independent variables were held constant in the model, CRP concentration over 161 mg/L on the day of admission was significantly associated with complications (adjusted odds ratio (OR_adj_): 3.56; 95% CI: [1.22, 10.40], *p* = 0.020). Age (OR_adj_: 1.03, 95% CI: [1.01, 1.06], *p* = 0.014), an unknown entry point (OR_adj_: 3.94, 95% CI: [1.33, 11.62], *p* = 0.013) and a entry point from an IMD (OR_adj_:4.58, 95% CI: [1.14, 18.35], *p* = 0.032) were also significantly associated with complications. Conversely, haemodialysis (OR_adj_: 0.09, 95% CI: [0.01, 0.71], *p* = 0.023) and an entry point from a DFU (OR_adj_: 0.12, 95% CI: [0.02, 0.77], *p* = 0.025) were inversely associated with complicated disease.

**Table 5:** Multiple logistic regression: potential risk factors for complications of *S. aureus* bacteraemia

Covariates [*n* = 139]	OR_adj_	95% CI	*p*-value	95% CI^a^	*p*-value
Age	1.03	(1.01, 1.06)	0.013	(1.01, 1.06)	0.014
Haemodialysis	0.09	(0.01, 0.97)	0.047	(0.01, 0.71)	0.023
Nausea	0.44	(0.18, 1.07)	0.070	(0.18, 1.07)	0.069
Diabetic foot ulcer entry point	0.12	(0.02, 0.62)	0.012	(0.02, 0.77)	0.025
Unknown entry point	3.94	(1.31, 11.84)	0.015	(1.33, 11.62)	0.013
Indwelling medical device entry point	4.58	(1.21, 17.26)	0.025	(1.14, 18.35)	0.032
CRP over 161 mg/L on day of admission	3.56	(1.29, 9.79)	0.014	(1.22, 10.40)	0.020
White blood cell count over 14.85 × 10^9^ cells/L on day of admission	2.93	(0.89, 9.69)	0.077	(0.93, 9.28)	0.067

*OR*_*ad*j_ Adjusted Odds Ratio

Post estimation tests: Hosmer-Lemeshow’s goodness of fit: chi2(8) = 5.12, *p* = 0.744, Area Under Curve (AUC) = 0.85, Correctly Classified = 79.1%

^a^Robust Standard Errors used to calculate 95% CIs

## Discussion

Current reported global mortality rates of SAB range between 10 and 35% [3, 5, 32, 33] and are related to complications of the infection. Identifying complications earlier may improve implementation of appropriate therapy, identify the need for additional interventions and reduce this mortality rate [34]. To that end, several attempts to identify risk factors for complications have been attempted [35–38]. These studies are varied in definitions, methodology and populations. They also often focus upon one specific complication. Predictive models have also been attempted [39–42], but focus upon predicting mortality, a specific complication, or are hospital-acquired infections and thus may not be generalizable to the increasingly prevalent community-acquired setting of infection.

To our knowledge, no study has focussed specifically upon community-associated SAB; the predominant cause in Australia. In this study, we aimed to identify risk factors for complications from demographics, self-reported symptoms, portal of entry, and microbiological profile of the bacteria and from routine biomarkers in SAB.

Complicated SAB represented 67.6% of the cohort, with the median age of the cohort 68.5 years (IQR:29.0). Age was significantly associated with complications and this is also reflected in other studies [5, 32, 36, 43].

Haemodialysis (OR_adj_: 0.09, 95% CI: [0.01, 0.71], *p* = 0.023) was significantly inversely associated with complications in SAB. This may be due to the increased medical attention received by those who are on haemodialysis. It may be also be due to the portal of entry from an infected line, with early removal associated with improved outcomes [44]. However, it must be noted that patients with haemodialysis have been previously associated with increased risk for infective endocarditis [8] in all-cause bacteraemia infections.

Significantly, portals of entry were strongly associated with complications in SAB. In this study, an unknown portal of entry was the most common entry point of infection. The higher prevalence of unknown entry point in this study when compared to others, may be due to our definition of portal of entry rather than the infective focus that other studies have used [3, 45].

An unknown portal of entry was significantly associated with complications (OR_adj_: 3.94, 95% CI: [1.33, 11.62], *p* = 0.013), as seen previously [46]. This may be due the increased risk of persistence due to the inability to identify the portal of entry and remove the infective focus, increasing the likelihood of *S. aureus* creating metastatic foci.

A portal of entry from an indwelling medical device (OR_adj_: 4.58, 95% CI: [1.14, 18.35], *p* = 0.025) was also significantly associated with complications. This may be due to the definition of complication which automatically categorises those with a prosthesis or cardiac device as a complicated case, regardless of whether the medical device is implicated in the infection [20]. Conversely, a portal of entry from a diabetic foot ulcer (OR_adj_: 0.12, 95% CI: [0.02, 0.77], *p* = 0.012) was inversely associated with complications. This may be due to increased management of diabetic foot ulcers but could also be due to the suppressed immune effects and reduced blood supply to the ulcer [47].

Investigation into predictive factors from routinely performed blood tests in a clinical setting showed three possible markers associated with complications in univariable analysis. Cut-off values showed high confidence intervals in white blood cell count and neutrophil cell count. This was due to the difference in numbers between the complicated and uncomplicated groups.

It was identified that a CRP concentration of over 161 mg/L on the day of admission was significantly associated with complications (OR_adj_: 3.56; 95% CI: [1.22, 10.40], *p* = 0.020). CRP is an acute phase protein of the pentraxin family. It is raised following an inflammatory injury and peaks the following day [48]. It aids in bacterial clearance by activating the classical pathway of the complement system through binding to Cq1 [49]. Interestingly, an inverse relationship has been observed in sepsis cases where a decreased expression of the HLA-DR marker on monocytes was associated with persistently increased CRP concentration over time [19]. CRP has been investigated as a predictive risk factor in adult SAB, with differing outcomes and study populations reflecting all different results. As seen in Table 6, prior studies are varied in terms of setting, population, outcome assessed, and potential cut-off value identified [3.0 mg/L to 850 mg/L]. Our study in comparison, is mid-range in terms of study number, has the benefit of being a prospective collection, and attempts to include all complications. Conversely, our study is set in a community-onset SAB population, limiting the generalisability of the findings to all SAB cases.

**Table 6:** Summary of C-reactive protein association studies with bacteraemia

StudyFirst Author	Setting	Population	Cut-off [mg/L]	Poor outcome	[Ref].
Horino	RetrospectiveSingle centre *n* = 73	AdultMSSA-SAB	> 30.0	Metastatic infection	[50]
Holmes	Prospective Multi-centre*n* = 222	Adult SAB	> 250.0	30-day Treatment failure	[32]
Pravin	ProspectiveCross-sectional Single centre*n* = 75	Neonates,All-causeSepsis symptoms	> 30.0	Sepsis	[51]
Bernstein	Retrospective, Single centre*n* = 109	Paediatric***S. aureus***Septic arthritis	> 13.7	MRSA Septic arthritis	[52]
Bouchard	Retrospective, Single centre*n* = 183	PaediatricAll-causeSeptic arthritis	< 20.0	Not reliable as a marker for clearance of infection	[53]
Tascini	RetrospectiveMulti-centre*n* = 236	AdultAll-cause Infective endocarditis	> 130.0> 850.0	***-S. aureus*** **i**nfective endocarditis-Death	[54]
Molkanen	ProspectiveMulti-centre*n* = 430	Adult SAB	Day 4: > 103.0Day 14: > 61.0Day of diagnosis: > 108.0Day 14: > 22.0	-death-death-deep infection-deep infection	[55]
Lin	RetrospectiveSingle centre*n* = 108	All ages,All-cause Osteomyelitis	> 5.0> 8.0	-Prosthesis related-osteomyelitis recurrence-Prosthesis unrelated-osteomyelitis recurrence	[56]
Chiappini	RetrospectiveSingle centre*n* = 121	PaediatricAll-causeAcute haematogenous osteomyelitis	> 10.0	Complicated acute haematogenous osteomyelitis	[57]
Tang	RetrospectiveSingle centre*n* = 825	AdultAll-cause bacteraemia	No cut-off	Distinguishes positive and negative cultures,Distinguishes between gram positive, negative and candidaDistinguishes between species	[58]
Garcia del Pozo	RetrospectiveSingle centre*n* = 116	AdultAll-causeLong boneosteomyelitis	NA	Relapse recurrent osteomyelitis	[59]
Botheras	ProspectiveSingle centre*n* = 158	Adult community-onset SAB	> 161.0	Complicated SAB	

CRP and full blood examinations are already routine blood immunoassays utilised in the diagnosis of infection and therefore have known parameters and remains stable when affected by delays into processing [60, 61]. Therefore, these assays could quickly and efficiently be implemented into a management guideline. CRP could be an additional tool for resource poor healthcare settings to manage the condition by prioritising those with high CRP to have further diagnostic tests to assess for complications. Furthermore, given the increasing positive data on CRP as a predictive marker, it should be highly considered as an accessory marker for triaging patients. Future studies can help confirm the most appropriate cut-off value.

It also highlights the potential for other biomarkers to be predictive. Other studies have found several including, procalcitonin, IL-6, IL-10, IL-1β, IL-17, TNFa and IFNy [62–67]. However, these markers are not routinely used in a clinical setting and to implement them into clinical practice would require significant additional resources.

There are several limitations to this study. Firstly, this study is from a single centre and the sample of patients is relatively modest. This limits the generalisability of the findings in other settings and potentially a loss of power to detect weaker associations that may exist. However, Pasco and colleagues demonstrated that the Geelong region population closely reflects the Australian population, through comparing the Barwon Statistical Division that represents Geelong and the national average, and showed the most divergent factor [Country of birth] differed by 9.5% [67]. Nonetheless, results of the study are promising, and we plan to extend this work to a larger study with multiple sites to further validate our model.

## Conclusion

In this study, we aimed to identify potential predictive factors of complications in adult community-associated SAB. This included a quantified cut-off predictive marker of any potential blood markers already used in the clinical setting. It was identified that a history of haemodialysis was protective against complications in this cohort. The importance of the portal of entry in the outcome of SAB was observed. An unknown entry point and an entry point originating from an IMD were both significantly associated with complications, whereas a portal of entry from a DFU was protective against complications. Most importantly, we identified that a CRP over 161 mg/L on the day of admission was significantly associated with complicated disease and might offer a useful predictive tool to aid clinicians in the management of adult cases of community-associated SAB.

## Data Availability

The datasets used and/or analysed during the current study are available from the corresponding author on reasonable request.

## Abbreviations

*S. aureus*: *Staphylococcus aureus*
SAB: *Staphylococcus aureus* bacteraemia
CRP: C-reactive protein
UHG: University Hospital Geelong
EMR: Electronic medical records
CACI: Charlson age comorbidity index
HIV: Human immunodeficiency virus
SSTI: Skin and soft tissue infection
IMD: Indwelling medical device
IDU: Injecting drug use
PSSA: Penicillin-sensitive *Staphylococcus aureus*
MSSA: Methicillin-sensitive *Staphylococcus aureus*
nm-MRSA: Non-multiple methicillin-resistant *Staphylococcus aureus*
MRSA: Methicillin-resistant *Staphylococcus aureus*
IQR: Interquartile range
ROC: Receiving operating curve
CI: Confidence interval
OR: Odds ratio
OR_adj_: Adjusted odds ratio
AUC: Area under the curve
IL: Inter-leukin
